# From neurotechnology to the classroom: the promise of brain–computer interfaces for training systems engineers

**DOI:** 10.3389/fnhum.2025.1733768

**Published:** 2026-01-07

**Authors:** Christian Nieves-Méndez

**Affiliations:** Facultad de Ciencias Agrarias, Universidad Agraria del Ecuador, Guayaquil, Ecuador

**Keywords:** brain–computer interfaces, neuro education, systems engineering, adaptive learning, ethical AI

## Abstract

This perspective article explores the transformative potential of brain–Computer Interfaces (BCI) in undergraduate systems engineering programs, a domain characterized by high attrition and a widening gap between rapid technological innovation and slower pedagogical change. I argue that BCI, by enabling real-time detection of cognitive states such as mental workload, attention, and frustration, can evolve from laboratory tools to central pedagogical instruments for adaptive, student-centered education. I review the state-of-the-art methods, which demonstrate the technical feasibility of low-cost electroencephalography (EEG) devices and machine learning algorithms that classify cognitive states with high accuracy in controlled settings. Building on this evidence, I outline concrete applications in three dimensions: formative assessment, dynamic curricular adaptation, and cognitive inclusion, with a specific emphasis on preventing dropout in foundational courses such as algorithms. I also examine ethical, technical, and pedagogical challenges, and propose a scalable, ethically grounded pilot model tailored for universities, particularly in Latin America. This study reports no empirical results. It synthesizes the existing evidence and proposes a roadmap for research and educational action.

## Introduction

Imagine a programming class in which the learning environment senses when a student is on the verge of abandoning a task due to frustration and automatically adapts to the difficulty, modality, or type of feedback. The signal for this adaptation does not come from clicks or response times. This is due to brain activity. Advances in BCI and real-time neural signal processing have made this scenario technically plausible, even with consumer-grade hardware and lightweight classification models (Sawangjai et al., 2020; Chen et al., 2023).

Systems engineering education faces well-documented headwinds: elevated attrition in technical courses, persistent difficulty in measuring conceptual understanding beyond standardized tests, and a widening mismatch between the speed of technological change and curricular responsiveness (Luckin et al., 2016). These problems directly affect workforce quality in fields such as artificial intelligence, cybersecurity, and software development, where success depends not only on technical skills, but also on cognitive resilience and self-regulation (Zawacki-Richter et al., 2019).

Currently, portable and relatively affordable EEG devices, combined with machine learning, can detect attention, workload, and fatigue with accuracies that often exceed 80 percent in controlled environments (Sawangjai et al., 2020; Chen et al., 2023). In education, BCI has been used to adapt learning games, monitor engagement in massive open online courses, and predict performance in real-time. However, most implementations remain confined to experimental contexts or primary and secondary levels and seldom target university-level technical disciplines (Wang et al., 2025).

This critical gap persisted. Pedagogical frameworks and empirical studies that integrate BCI into university-level systems engineering are lacking, where highly abstract content in algorithms, computer architecture, and computational logic generates intense cognitive demands and spikes of frustration that traditional behavioral metrics fail to capture (Medeiros et al., 2021). Ethical and curricular discussions on BCI at this level are only beginning (Ienca et al., 2018).

Our position is that BCI should not be regarded as a laboratory curiosity. These are emerging pedagogical tools that can help transform engineering education by grounding instructional decisions in the learner’s actual cognitive state rather than in surface behavior.

This article focuses on the pedagogical potential of BCI in university systems engineering programs. I do not pursue clinical or therapeutic applications, nor do I delve into advanced hardware design. First, I review the state of the art, then propose viable applications for engineering education, analyze key challenges, and conclude with a pilot model for implementation in university settings.

### State of the art: from cognitive neuroscience to educational engineering

The convergence of neurotechnology and pedagogy has given rise to a new interdisciplinary space that I term computational neuroeducation. Its goal is to optimize learning by bringing objective measures of brain processes into educational decision-making. Recent studies have shown that consumer-grade EEG devices paired with deep learning or other classification approaches can separate attention and cognitive load states with average accuracies of approximately 85 percent in simulated educational contexts (Sawangjai et al., 2020; Chen et al., 2023). In programming education, researchers have begun correlating beta and theta patterns with moments of logical impasse and conceptual insight, which opens the door to timely pedagogical interventions tailored to cognitive moments rather than to a fixed lesson plan (Medeiros et al., 2021).

Despite this promise, much of the work remains experimental, and few studies have attempted to examine the semantic and syntactic complexity inherent in data structures, algorithms, or software architecture courses (Beauchemin et al., 2024). I read this limitation as primarily pedagogical, not technical. A principled translation layer that maps neurophysiological signals to teaching actions that instructors can implement in context is missing. In other words, neuropedagogical indicators that carry actionable meaning for course design and classroom orchestration are needed.

In addition to outlining viable micro-interventions, it is important to note which practices should be avoided. Neurophysiological signals such as EEG-based workload or attention estimates should not be treated as deterministic triggers for automated pedagogical actions. For example, adapting the difficulty of a task solely because a model detects “low attention” risks misinterpreting transient noise as cognitive meaning and undermines student autonomy. Similarly, continuous monitoring without explicit consent or intrusive feedback (e.g., real-time alerts labeling students as disengaged) can generate anxiety and erode trust. At the opposite end of the spectrum, macro-interventions involve structural changes to the learning environment, such as redesigning curricular pathways based on optional self-reflection supported by non-invasive neurofeedback, or creating laboratory experiences in which BCI signals help students analyze their cognitive strategies during complex problem-solving rather than serving evaluative purposes. These broader interventions prioritize ethical use, learner agency, and metacognitive development.

I saw two immediate opportunities. The first is to articulate the cognitive constructs that matter most in systems engineering courses, such as bottleneck detection during recursive debugging or frontocortical activation patterns associated with combinatorial optimization. The second is to link these constructs to micro-interventions that instructors can deploy during practice, such as altering the modality of an explanation, adjusting the scaffolding of a proof, or pacing the introduction of new symbols.

### Viable applications in systems engineering programs

I identified three domains in which BCI can add value without asking instructors to become neuroscientists: formative assessment, dynamic curricular adaptation, and cognitive inclusion.

#### Formative assessment

Continuous measures of workload, attention, and frustration can augment low-resolution behavioral indicators and provide a richer picture of where and why students struggle. Preliminary research suggests that monitoring cognitive load using BCI allows instructors or intelligent tutoring systems to adjust the complexity of programming exercises during practice, which reduces frustration and increases persistence among novice students (Yoo et al., 2023). In lab-like environments, interactive tutorials that shift modalities based on real-time attention signals have yielded gains in conceptual retention relative to controls (Papakostas et al., 2025). These results resonate with broader evidence on the dynamics of affect during complex learning, where timely support can prevent maladaptive spirals (D’Mello and Graesser, 2012).

#### Dynamic curricular adaptation

The promise of BCI is not to replace instructors, but to provide them with a cognitive radar that sharpens instructional timing. In the earliest algorithms and data structure courses, I recommend small, instructor-in-the-loop prototypes that link specific neuropedagogical indicators to defined teaching moves. If a pattern associated with cognitive bottlenecks emerges during recursion or pointer arithmetic, the system can switch examples, activate a hint, or adjust the abstraction level, while preserving the instructor’s agency. Early work indicates that such adaptations can improve conceptual outcomes by meaningful margins, including gains in retention of up to 30 percent, while reducing cognitive overload (Yoo et al., 2023; Papakostas et al., 2025).

#### Cognitive inclusion

BCI-mediated neurofeedback can help students build self-regulation and emotional resilience, two skills that matter for sustained debugging and problem-solving in professional practice (Ienca et al., 2018). Instead of pathologizing variability in cognitive profiles, I propose using BCI signals to personalize supports that help diverse learners to remain engaged with challenging materials. This approach aligns with calls in educational technology to balance promises with a realistic appraisal of pitfalls and variability across learners (Tinga et al., 2019).

The near-term gains are likely to be largest in high-attrition courses, where mental states correlate strongly with specific learning milestones, and where small timing adjustments can prevent withdrawal. Algorithms, data structures, and low-level programming are natural starting points.

### Ethical, technical, and pedagogical challenges

BCI in university classrooms raises issues that cannot be solved using hardware alone. These are not obstacles to be waved away, but the design requirements to be addressed systematically.

#### Ethical considerations

Brain data is among the most intimate forms of personal information. Their collection in educational contexts raises questions about neuronal privacy, informed consent, data minimization, and the risk of cognitive profiling (Ienca et al., 2018). I endorse an ethics-by-design approach anchored in strong consent procedures, clear purpose limitations, strict anonymization or pseudonymisation, and independent review. Alignment with emerging global guidance on AI and education can provide a baseline for institutional governance (UNESCO, 2023).

#### Technical constraints

Consumer EEG devices have improved; however, they remain sensitive to environmental noise, motion artifacts, and interindividual variability. Calibration is often required for a stable classification performance, which complicates scaling to large classes (Sawangjai et al., 2020). These constraints argue for small-cohort pilots, robust signal preprocessing pipelines, and conservative claims. In our view, the bar for classroom adoption is not the elimination of noise but the demonstration that, despite noise, the system produces pedagogically meaningful signals with acceptable reliability.

#### Pedagogical readiness

Most instructors are not trained to interpret neurocognitive metrics or translate them into teaching strategies. Without support, a BCI can add friction rather than a value. Therefore, I recommend targeted faculty development and the co-design of dashboards that present a small set of indicators in clear instructional language. The goal is not to turn instructors into technicians, but to give them actionable insight at a glance (Luckin et al., 2016; Tinga et al., 2019).

These challenges should be addressed collaboratively by neuroscientists, engineers, educators and students. No single perspective suffices.

### Toward a pilot model for engineering universities

To move from the laboratory to the classroom, I propose a three-phase pilot model that balances technical feasibility with ethical and pedagogical integrity. Unlike prior work that focuses primarily on quantifying frustration, predicting persistence, or modeling dropout risk, the proposed three-phase pilot model aims to make invisible cognitive states pedagogically meaningful while preserving student autonomy and instructor agency. The model does not seek to optimize performance through automated adaptations; instead, it is grounded in an ethics-by-design framework in which BCI signals serve as prompts for empathetic, context-sensitive instructional decisions. Its contribution lies in positioning neurotechnology as a humanizing tool—one that reveals internal cognitive dynamics during high-attrition tasks and supports reflective decision-making by both learners and instructors. This emphasis on ethical governance, interpretability, and instructor-in-the-loop design differentiates the model from existing frustration- or retention-focused approaches.

#### Phase 1: identify and validate course-specific indicators

Beginning with a small cohort of 20–30 students in a high-attrition course. Collect EEG data during authentic programming tasks to train classifiers for states that matter instructionally such as cognitive bottlenecks during recursion. The literature suggests that even with four frontal electrodes (Fp1, Fp2, F7, F8) and lightweight models, such as Support Vector Machines or Random Forests, it is possible to detect relevant patterns with F1 scores above 0.78 (Medeiros et al., 2021; Yoo et al., 2023). Document both model performance and pedagogical interpretability of the signals.

#### Phase 2: integrate with existing platforms

The BCI classifier was connected to learning management systems and development environments through APIs. I envision simple triggers that adjust modality, pacing, or scaffolding in environments, such as Moodle or Visual Studio Code. The instructor-in-the-loop design should remain at default. The system can propose an adaptation and the instructor can accept, modify, or ignore it.

#### Phase 3: pedagogical evaluation with controls

The purpose of the Phase 3 evaluation is not to increase students’ speed or accuracy simply by providing timely machine-generated prompts. Rather, the goal is to understand whether neurocognitively informed interventions help students develop metacognitive awareness during complex problem-solving. When learners encounter a cognitive impasse, such as during recursion or debugging, the relevant question becomes: Is the difficulty due to cognitive load, the absence of a mental model, or emotional factors such as anxiety? A well-designed BCI system can make this internal dialogue more visible, allowing students to reflect on their strategies and choose whether to persist, request help, or change approaches. In this phase, instructors use BCI-derived indicators as interpretive cues rather than prescriptions, ensuring that pedagogical interventions cultivate autonomy, self-regulation, and conceptual understanding rather than dependency on automated feedback (Papakostas et al., 2025).

All phases operate under robust ethical governance. Secure informed consent, apply strict anonymization, and convene an independent review committee. Align protocols with international guidance for AI in education (UNESCO, 2023). Latin American universities, with their curricular flexibility and close instructor–student relationships, are well positioned to conduct such pilots and generate context-sensitive evidence before scaling.

## Discussion

Two conclusions emerged from the literature that motivated our proposal. First, technical feasibility is no longer the primary question. Studies show that consumer EEG devices combined with lightweight classifiers can detect cognitive states that are instructionally relevant, with accuracies exceeding 80 percent and F1 scores above 0.78 (Sawangjai et al., 2020; Medeiros et al., 2021). These signals reach a layer of cognitive experience that behavioral logs cannot capture in real-time. Second, early applications of neuro-adaptive systems in computing education, although still limited in scope, demonstrate pedagogically meaningful effects. Adaptive tutorials and BCI-guided interventions reduce frustration, increase persistence, and improve conceptual retention by significant margins, relative to controls (Papakostas et al., 2025). These outcomes compare favorably with purely behavioral learning analytics, which often provide incomplete pictures of learners’ internal states (Medeiros et al., 2021).

Why are these findings promising for systems engineering programs? This is because the conceptual demands of algorithms and data structures are intrinsically high. The cognitive peaks and troughs students experience are largely internal. Students can look focused while experiencing collapse in their working memory. Traditional metrics are not currently available. BCI can be invisible and enable proactive, rather than reactive, support. Detecting a theta-dominant pattern associated with a logical impasse during recursion, for example, can trigger a strategic hint or change in representational modality before disengagement takes root (Medeiros et al., 2021). Far from encouraging dependence, well-designed neurofeedback can cultivate self-awareness and self-regulation, which are essential for professional software practices (Ienca et al., 2018).

However, this does not mean that classroom-scale adoption is imminent. The evidence base is preliminary and the risks are real. However, technical noise and individual variability remain formidable. Faculty capacity is limited. Ethical frameworks continue to evolve. Thus, our stance is pragmatic. I advocate for small, carefully governed pilots that measure both learning outcomes and learners’ well-being. I prefer interpretable indicators and instructor agencies to black box automation. I value the negative and null results as much as the positive results. In short, I argue for a disciplined optimism.

Once small, well-governed pilots are validated, several avenues for future research emerge. First, scalability should focus on depth rather than size: replicating pilots in other high-demand courses such as computer architecture or cybersecurity could help identify discipline-specific cognitive profiles and inform the design of more human-centered learning trajectories. Second, institutions could develop micro-credential programs in neurocognitive literacy to strengthen instructors’ ability to interpret brain-signal indicators responsibly. Third, BCI systems may be integrated into reflective learning practices, for example, as tools that help students analyze their cognitive strategies within portfolios or authentic assessment frameworks. Finally, a coordinated network of Latin American universities could generate contextualized evidence, valuing negative or null results as part of rigorous neuropedagogical inquiry and ensuring that innovations reflect local realities rather than imposed external models.

### A research and action agenda

I see five near-term priorities in this field. First, institutions should design and validate a concise set of neuropedagogical indicators that genuinely matter in systems engineering courses and then test their relationship to learning processes through controlled classroom studies with cohorts of 20 to 30 students. Second, teams should build instructor-centered dashboards that translate brain-signal classifications into clear instructional prompts and evaluate their usability, as well as their effect on instructors’ sense of self-efficacy. Third, programs must embed ethics by design, with robust procedures for informed consent, data minimization, anonymization, and independent review in alignment with global guidance on AI in education (UNESCO, 2023). Fourth, universities should launch short practical courses in neurocognitive literacy so that instructors can interpret a small set of indicators and plan appropriate instructional responses. Fifth, stakeholders should establish a consortium of Latin American universities to coordinate pilots, pool anonymized datasets, and examine contextual variables that influence generalizability.

## Conclusion

The promise of BCI in systems engineering education is less focused on technology than pedagogy. It is about building learning environments that can sense the learner, recognize invisible cognitive effort, and respond with intelligent empathy. By integrating insights from cognitive neuroscience into everyday instruction, I can create classrooms that are more adaptive, humane, and more effective. Learners’ dignity resides in their subjective experience. Our responsibility as educators is to make that experience visible enough to support, understandable enough to respect, and malleable enough to improve it. BCI can help us achieve this goal. The path forward is not a leap-to-mass deployment, but a series of careful, ethical, and pedagogically grounded pilots. Done well, they will amplify the instructor’s capacity to teach with the brain in mind, not only the brain.

## Data Availability

The original contributions presented in the study are included in the article/supplementary material, further inquiries can be directed to the corresponding author.
